# Sever erythema multiforme post-COVID-19 moderna vaccine: Case report and literature review

**DOI:** 10.1016/j.amsu.2022.104461

**Published:** 2022-08-20

**Authors:** Abdalla Fadul, El Mustafa Abdalla, M. Musa, Abdulrhman AL-Mashdali, Ahmed EL. Mudathir Osman, Elabbass Abdelmahuod

**Affiliations:** Department of Internal Medicine, Hamad Medical Corporation, Doha, Qatar

**Keywords:** COVID-19, Vaccine, Moderna, Erythema multiform, EM

## Abstract

**Introduction:**

Although the COVID-19 Vaccine usually causes a few non-serious side effects, serious ones such as Erythema multiforme recently has been linked to it.

**Case presentation:**

Our patient presented with severe skin reaction one day post-Covid-19 Moderna vaccine diagnosed as erythema multiforme proven by skin biopsy that responded well to steroids.

**Discussion:**

Erythema multiform major, an immune-mediated cutaneous reaction to infections or drugs involving the oral cavity, should be considered a possible adverse effect of numerous vaccinations, including SARSCoV2. Correct patient history gathering enables early detection and successful medical therapy with oral corticosteroids.

Furthermore, the disease's rarity makes establishing a causative link difficult. However, because we are still learning about the innovative antiSARSCoV2 vaccines, it is crucial to be cautious of the potential cutaneous adverse responses.

**Conclusion:**

Despite being rare, life-threatening adverse reactions can occur post-COVID-19 Vaccination.

## Highlights

• Covid-19 Moderna vaccine is approved for vaccination against CVOID-19 infection.
• Adverse skin reactions ranging from mild to Sever can occur post-Moderna vaccine.
• Erythema multiforme Is a rare but serious skin reaction.
• Moderna vaccine can lead to life-threatening complications in a few selected patients.

## 1 Introduction

Due to the recent COVID19 pandemic, the use of mRNA vaccines Pfizer–BioNTech and Moderna has lately been expanded, allowing the identification of numerous cutaneous side effects associated with this vaccination [1]. Although erythema multiforme (EM) is a known unusual side effect of many different vaccinations, few reports relate this reaction to mRNA vaccines. Here, we present a typical EM case that occurred quickly after the first dosage of the Moderna vaccine and had no other apparent cause [2].

Erythema multiforme is an immune-mediated chronic inflammatory illness affecting the skin and mucous membranes, usually associated with herpes simplex virus (HSV) infection [3]. However, Other causes have been identified, including various infectious organisms, medications, vaccines, and even internal illnesses. And recently has been linked to the COVID-19 vaccine [4].

The early EM lesions have a target look, with a dusky core section surrounded by a dark red inflammatory zone and another lighter ring on the perimeter. Geographic, polycyclic, and annular geometries are standard in EM lesions. The oral, ocular, or vaginal mucosa can be affected in extreme instances [5]. Atypical EM clinical patterns, such as an odd distribution or an absence of the target appearance, are frequently referred to as EM-like eruptions. Inflammatory perivascular and interface infiltration, hyperkeratosis, mucinosis, and acanthosis are some histological features that can be seen [6].

## 2 Case presentation

**Figure 1:**
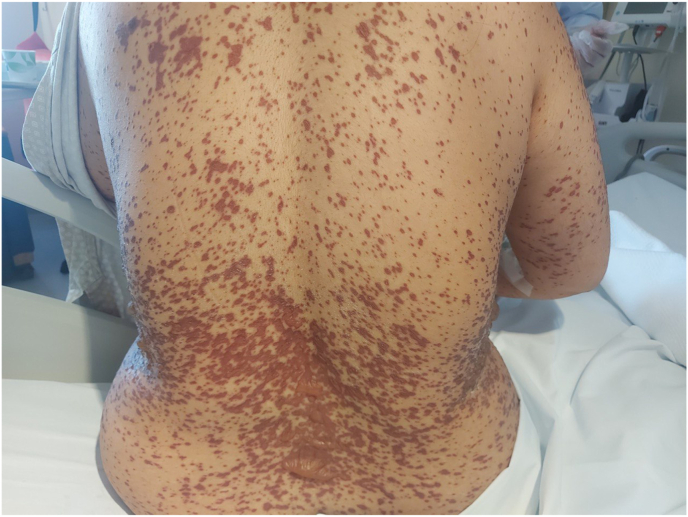
Multiple dark erythematous, maculopapular lesions on the posterior trunk.

**Figure 2:**
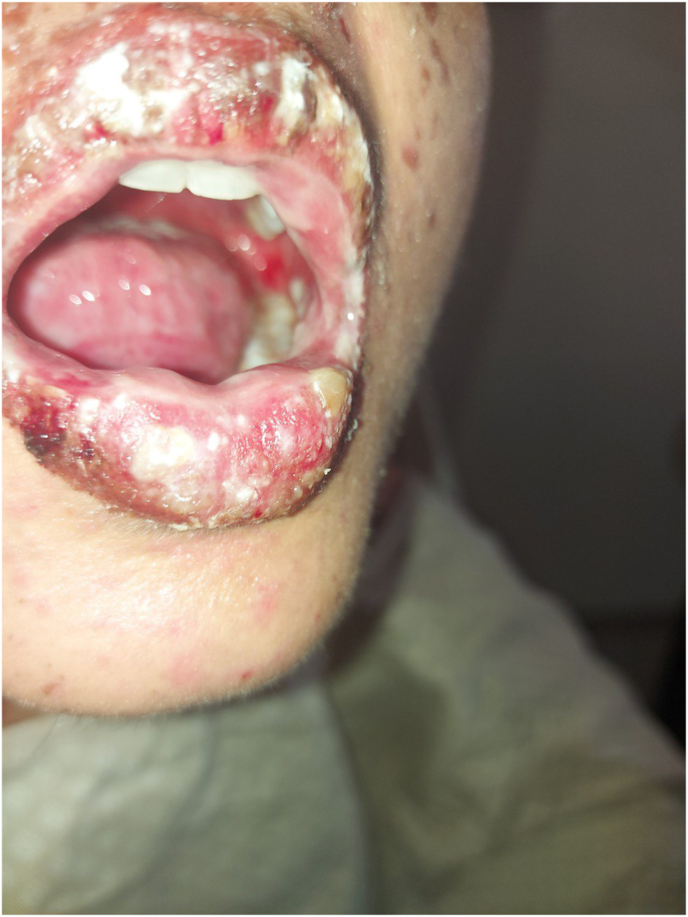
Many blisters and erosions on the lips, hard and soft palate, and buccal mucosa on both sides.

**Figure 3:**
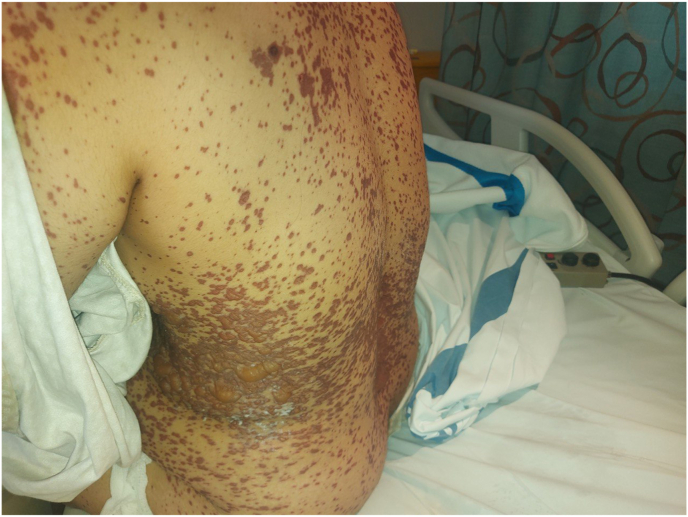
Multiple dark erythematous, maculopapular lesions on the lateral and posterior trunk, along with many blisters and erosions

36-year-old female, previously healthy, 24 hours post the first dose of covid-19 moderna vaccine, she developed a sore throat, painful mouth ulcers, bilateral red and itchy eyes, and itchy skin lesions that started on her chest, back, and genital area. She quickly spread to the rest of her body, becoming intensely itchy, See Figure 1, Figure 2, Figure 3.

When she arrived at the Emergency Department, she was vitally stable, afebrile, with unremarkable other vital signs. Her initial physical examination showed Multiple dark erythematous, maculopapular lesions on the face, anterior and posterior trunk, thighs, and upper limbs, sparing the palms and soles. There were also follicular papules on the mons pubis (genital area) along with many painful blisters and erosions on the lips, hard and soft palate, and buccal mucosa on both sides with congestion in both eyes.

Initial laboratory tests were normal, including a complete blood count, a renal function test, and a liver function test. Her C-reactive protein was 31 mg/L (normal range< 5 mg/L), and a nasopharyngeal swab for SARSCoV2 was negative. Chlamydia pneumoniae (IgG and IgM), Mycoplasma pneumoniae (IgG and IgM), herpesviruses 1 and 2, measles (IgM and IgG), and varicella (IgM and IgG), *T. pallidum*, hepatitis C, and hepatitis B were all found to be negative on serological testing.

Based on clinical presentation and medical history, a diagnosis of vaccine-related erythema multiforme was made, which was subsequently confirmed by skin biopsy, and IV dexamethasone 8 mg, IVIG to 0.5g/kg, as well as fluid supplementation, were started.

She finished five days of IVIG and began tapering the dose of steroids. Her clinical picture gradually improved, with significant improvement in her rash and oral intake.

She was safely discharged with instructions to skip the second dose of covid vaccination. She was later seen at a dermatology clinic, where she was entirely asymptomatic and in good condition.

## 3 Discussion

As the worldwide immunization effort increases and advances, several dermatological side effects have been discovered. Many of them have been documented following the SARS-CoV-2 immunization, ranging from mild urticarial and local injection-site responses to multiple bullous eruptions.

EM is a hypersensitive reaction to a variety of antigenic stimuli that appears as “targets lesions” on the skin and mucosa. Infections have been shown to be the main cause in the vast majority of cases, with HSV types 1 and 2 and Mycoplasma pneumoniae being the most prevalent etiologies [7].

Cell-mediated immunity is responsible for the abnormal degradation of epithelial cells. Lymphocyte infiltration at the dermo-epidermal junction caused inflammation and eventually death of epithelial cells. The majority of the lymphocytes are CD8 T cells and macrophages The punch biopsy is the standard method for establishing an EM diagnosis. The lesion biopsy will reveal apoptosis and degeneration of basal keratinocytes, edema, and blisters on cellular levels. In our case, necrotic keratocytes with perivascular lymphocytic infiltration were found in the skin sample, confirming the diagnosis [8].

The skin rash is symmetrical in younger individuals, but the trunk is frequently spared. The clinical presentation is frequently atypical [6]. In our case, the patient had discrete skin lesions affecting the anterior and posterior trunk but sparing the palms and soles.

Many recent studies have shown EM as an uncommon cutaneous adverse effect of the COVID19 vaccination. In addition to our patient, three cases of EM-associated with doses of mRNA127 (Moderna COVID19 vaccine) were discovered among 414 health professionals who experienced cutaneous reactions to COVID19 vaccinations [9].

Other cases of EM associated with the COVID19 vaccine have also been documented. The first is a 91-year-old woman who developed widespread skin lesions on her back and limbs. A biopsy of the skin lesion confirmed her diagnosis ten days after the (Pfizer COVID19 vaccination). 1 The second patient is a 38-year-old male who was sent to a dermatological clinic with extensive targetoid lesions after receiving the (Pfizer COVID19 vaccination); his diagnosis was confirmed by an excisional biopsy [10].

Additional studies are needed to understand the specific mechanism through which SARS-CoV-2 is linked to EM development, especially now that COVID-19 vaccination is ongoing and more data relating to EM and COVID-19 immunization is becoming available.

The work has been reported in line with the SCARE 2020 criteria [11].

## 4 Conclusion

Finally, cutaneous adverse responses to the SARSCoV2 vaccine were extremely rare, minor, and generally resolved spontaneously. We should provide advance notice and reassure the vaccinator. Nonetheless, these and other adverse outcomes should not deter people from getting vaccinated against a potentially fatal illness.

## Provenance and peer review

Not commissioned, externally peer reviewed.

## Availability of data and materials

The datasets used and/or analyzed during the current study are available from the corresponding author on reasonable request.

## Declaration of competing interest

The authors have no competing of interest to declare.

## Role of sponsors

No role in our study.

## Ethical approval

The case report was approved by Hamad Medical Corporation Medical Research Centre.

## Sources of funding

Open access funding was provided by Qatar National Library (QNL).

## Author statement contribution

Abdalla Fadul identified the case, reviewed the literature, and wrote the manuscript. ELMustafa Abdalla is the corresponding author who helped in manuscript writing, doing a review for literature.

Muzamil Musa, Abdulrhman AL-Mashdali, Ahmed EL Mudathir Osman, and Elabbas Abdelmahmoud helped in identifying the case, reviewing the literature, and doing the final review and approval for the manuscript.

## Registration of research studies

Not required.

## Guarantor

ELMustafa Abdalla.

## Reference number

MRC-04-22-241.

## Consent

Written informed consent was obtained from the patient for publication of this case report and the accompanying image. A copy of the written consent is available for review by the Editor-in-Chief upon request.

## Declaration of competing interest

The authors have no competing of interest to declare.
